# Clinical evaluation of the Asian proximal femur intramedullary nail antirotation system (PFNA-II) for treatment of intertrochanteric fractures

**DOI:** 10.1186/s13018-014-0112-5

**Published:** 2014-11-13

**Authors:** MingHui Li, Lei Wu, Yang Liu, CaiMing Wang

**Affiliations:** Department of Orthopaedics, Fifth Hospital of Wuhan City (Second Affiliated Hospital of Jianghan University), Wuhan, 430050 Hubei Province China

## Abstract

**Background:**

The preferred treatment of intertrochanteric fractures in aged patients is controversial. The purpose of the present study was to evaluate the outcomes of the Asian proximal femur intramedullary nail antirotation system (PFNA-II) for stabilization of such fractures.

**Methods:**

The PFNA-II was used to treat intertrochanteric fractures in 163 elderly patients from March 2010 to March 2013. The patients comprised 69 men and 94 women with a mean age of 74.7 ± 13.0 years. All fractures were classified by the Orthopaedic Trauma Association classification system; 53, 83, and 27 fractures were classified as 31A1, 31A2, and 31A3, respectively. We statistically evaluated the intraoperative blood loss, operation time, incision length, X-ray exposure time, and postoperative outcomes. Patients were followed up for a mean of 15.2 months (range, 10–24 months). Functional outcomes were assessed according to the Harris hip scoring system.

**Results:**

Statistical analysis revealed an average operation time of 45.7 min (range, 35–110 min), average intraoperative blood loss of 115.2 mL (range, 65–430 mL), X-ray exposure time of 2.7 ± 1.4 s (range, 2–6 s), and total incision length of 6.5 ± 2.2 cm (range, 5.5–13.0 cm). Patients were followed up for a mean of 14.5 months (range, 10–24 months). The neck shaft angle was 134° ±15° (range, 115°–150°), and the fracture healing time was 14.0 ± 2.5 weeks (range, 11–19 weeks). The Harris hip score was 85.6 ± 17.5 points (range, 65–100 points) and included 41 excellent cases (25.15%), 92 good cases (56.44%), 26 moderate cases (15.95%), and 4 poor cases (2.45%) for a positive outcome rate of 81.60%. There were no varus hip deformities, screw cutouts, or femoral shaft fractures. Fourteen patients had thigh pain (9.82%), and five had inner thigh pain (3.07%); seven had more severe pain that was improved by physical therapy.

**Conclusion:**

PFNA-II has the advantages of a simple operation, few complications, and clinical efficacy for the treatment of intertrochanteric fractures. However, evaluation of its long-term efficacy and risk of other complications requires a large-sample, multicenter observational study.

## Introduction

The incidence of intertrochanteric fractures is increasing with the aging of society. Treatment of intertrochanteric fractures in elderly patients is a huge challenge for many trauma surgeons, mainly because many such patients have severe osteoporosis and medical disorders that increase the risks associated with surgery and anesthesia. Therefore, choosing the optimal fixation method and instrumentation is essential for a positive therapeutic effect.

In 2003, the proximal femoral nail antirotation (PFNA) system was put into clinical use by the Association for Osteosynthesis/Association for the Study of Internal Fixation (AO/ASIF). Although the use of the PFNA for treatment of proximal femoral fractures has achieved good clinical efficacy, a series of complications in Asian patients has been reported in the literature. In 2009, the AO/ASIF organization established the characteristics of the PFNA for Asian patients (PFNA-II). Few published reports have systematically assessed the role of the PFNA-II in the stabilization of intertrochanteric fractures. From March 2010 to March 2013, the PFNA-II was applied in the treatment of intertrochanteric fractures in 163 elderly patients.

## Materials and methods

### Materials

In total, 207 patients aged >65 years with peritrochanteric femoral fractures were treated with the PFNA-II at the Fifth Hospital of Wuhan City from March 2010 to March 2013 and enrolled in this study. After exclusion of patients with pathological fractures, trauma, and open injury, 163 patients were included in the statistical analysis. The patients comprised 69 men and 94 women with a mean age of 74.7 years (range, 65–95 years). This study was approved by the ethics committee of our hospital, and informed consents were obtained from patients or their authorized persons.

According to the Orthopaedic Trauma Association (OTA) classification system, 53 (32.52%) fractures were classified as 31A1, 83 (50.92%) as 31A2, and 27 (16.56%) as 31A3. Accidental falls occurred in 117 cases and traffic accident injuries in 46. All patients had closed fractures (multiple injuries were not selected). In total, 122 patients had more than one type of disease: 82 had hypertension, 32 had cerebral infarction, 37 had type II diabetes, and 32 had renal insufficiency; 7 patients underwent surgical operations on the contralateral femur. According to the American Society of Anesthesiologists (ASA) scoring system, 46, 85, and 32 patients had an ASA status of I, II, and III, respectively (Table 1).

**Table 1:** **Preoperative patient data**

**Variables**	**Value**
Patients	163
Female	94
Male	69
Mean age, years (SD)	74.7 ± 13.0
OTA classification	
31A1 (%)	53 (32.5)
31A2 (%)	83 (50.9)
31A3 (%)	27 (16.6)
Mechanism of injury	
Domestic fall	117
Traffic accidents	46
ASA classification	
1	46
2	85
3	32
Comorbidity	
Hypertension and cardiovascular diseases	82
Sequelae of cerebral infarction	32
Diabetes mellitus	37
Chronic renal insufficiency	32

### Methods

#### Perioperative examination and treatment

After admission to the hospital, each patient’s limb was elevated on a Blanc frame and abducted. If the patient was expected to undergo surgery within 7 days, the limb bones underwent only skin traction, not skeletal traction.

Elderly patients often have various medical disorders, and perioperative examination and treatment are thus very important. For patients aged >70 years, we routinely performed ultrasound examinations of the heart, vertebral arteries, and lower extremity vasculature and assess cardiac function, excluding patients with vertebral artery and deep vein thrombosis. For patients with respiratory infections, we administered preoperative antibiotics to maintain the patient’s hemogram and C-reactive protein level within acceptable limits. The blood glucose level of patients with diabetes was monitored seven times daily, excluding some patients with mild diabetes. Insulin therapy was administered to most patients, and the daily fasting and postprandial glucose levels did not exceed 10 mmol/L. A first-generation cephalosporin antibiotic was administered to prevent infection 30 min before surgery and again within 24 h; for selected patients (such as those with diabetes), antibiotic treatment was extended to 48 h.

#### Preoperative preparation

Before surgery, all patients underwent lateral femoral X-rays, estimation of the size of the canal, and determination of the nail diameter and length. For patients with a shorter height, we carefully considered whether or not to use the PFNA-II. For taller patients, patients with Evans type IV fractures and patients whose fracture line extended below the lesser trochanter, a 200- or 240-mm nail length was considered to increase the stability of the fixation.

#### Surgical methods

All operations were completed by an experienced orthopedic surgeon. The first three operations were not included in the study analysis to eliminate the effect of the learning curve.

The patient was placed in the supine position on an extension table. The hip and knee of the healthy limb were flexed and abducted to facilitate lateral C-arm fluoroscopy. A single pad was placed under the hip to raise the limb by 5 cm, and the limb was adducted about 10°. The fracture was reset under C-arm X-ray fluoroscopy. An approximately 4- to 7-cm proximal and longitudinal incision was made through the fascia and gluteus to expose the tip of the greater trochanter. The proximal canal was then opened by evenly applied force to avoid breakage of the greater trochanter. After insertion of a reamed nail, fluoroscopy was performed to evaluate the fracture situation. By the anteroposterior C-arm fluoroscopy, the guide pin is located in 1/3 of the femoral neck and located central of the femoral neck by lateral fluoroscopy. If the position of the guide pin was poor, then the pin should be adjusted to the correct position, but repeated adjustments should be avoided. For unstable Evans type III or IV fractures, in order to prevent the spiral area of the femoral head when the spiral blade was being pounded, we can insert it into an antirotation guide pin.

#### Postoperative rehabilitation

The first day after the isometric quadriceps and ankle pump exercises had been performed, the first 2 days of hip and knee flexion and extension exercises were initiated and the patients’ X-rays were reviewed. The mean time of part load of 31A1 fractures was 9.2 days (range, 5–14 days), that of 31A2 fractures was 21.7 days (range, 18–35 days), and that of 31A3 fractures was 41 days (range, 35–72 days). The patient was allowed to bear full weight after the disappearance of the fracture line on X-rays.

#### Postoperative follow-up and treatment evaluation

The operative time was defined as the duration of time from the start of closed reduction to completion of wound suturing. The operative time, fluoroscopy time, blood loss during surgery, and load time after the operation were evaluated by retrospective statistical analysis. The average follow-up period was 14.5 ± 6.2 months (range, 10–24 months). Clinical and radiographic examinations were performed at 4 and 6 weeks and at 3, 6, 12, and 18 months postoperatively. A Harris hip score of 90 to 100 was considered excellent, 80 to 89 was considered good, 70 to 79 was considered moderate, and ≤69 was considered poor.

#### Ethical considerations

All patients gave informed consent prior to their inclusion in the study. All human studies were approved by the Ethics Committee of the Second Affiliated Hospital of Jianghan University and were performed in accordance with ethical standards.

## Results

In total, 163 patients underwent either closed reduction (*n* =131) or limited open reduction (*n* =32). The average time from injury to surgery was 3.7 days (range, 2–14 days). The mean operative time was 45.7 min (range, 35–110 min), intraoperative blood loss was 115.2 mL (range, 65–430 mL), number of intraoperative C-arm fluoroscopy procedures was 2.7 ± 1.4 (range, 2–6), and total incision length was 6.5 ± 2.2 cm (range, 5.5–13.0 cm). The PFNA-II is available in three different lengths: the standard length (240 mm) was used in 22 patients, a length of 200 mm was used in 92 patients, and a very short length (170 mm) was used in 49 patients. The PFNA-II is also available in three different diameters: 9 mm was used in 42 patients, 10 mm was used in 93 patients, and 11 mm was used in 28 patients. Because patients who sustained traffic accident injuries were hospitalized for a longer period of time, statistical evaluation of the hospital stay was not performed. All patients were followed up for 14.5 ± 6.2 months (range, 10–24 months).

X-ray evaluation showed a neck-shaft angle of 134° ±15° (range, 115°–150°). Postoperatively, patients with type 31A1 fractures had an average loading time of 11.2 days (range, 7–16 days), those with type 31A2 fractures had an average loading time of 21.7 days (range, 18–35 days), and those with 31A3 fractures had an average loading time of 41.6 days (range, 35–72 days). The fracture healing time averaged 14.0 ± 2.5 weeks (range, 11–19 weeks). No patients exhibited postoperative nonunion, varus, or nail fracture. One patient developed reamer cutout into the acetabulum, and three developed reamer exit. Sixteen patients developed outer thigh pain (9.82%), and five developed inner thigh pain (3.07%); after the administration of nonsteroidal anti-inflammatory drugs (NSAIDs) and the performance of physical therapy, seven patients with severe pain experienced improvement. After the last follow-up, the mean Harris hip score was 85.6 ± 17.5 (range, 65–100); the score was excellent in 41 patients (25.15%), good in 92 (56.44%), moderate in 26 (15.95%), and poor in 4 (2.45%) for a positive outcome rate of 81.90% (Tables 2 and 3).

**Table 2:** **Intraoperative, perioperative, and postoperative clinical data**

**Variables**	**Value**
Closed reduction	131
Limited open reduction	32
Average time from injury to surgery (day)	3.7 (2–14)
Operation time (min)	45.7 (35–110)
Intraoperative blood loss (mL)	115.2 (65–430)
Intraoperative fluoroscopy times	2.7 (2–6)
Incision total length (cm)	6.5 (5.5–13)
Neck-shaft angle (°)	134 (115–150)
Fracture healing time (weeks)	14 (11–19)
Diameter of the nail (mm)	
9	42
10	93
11	28
Length of the nail (mm)	
170	49
200	92
240	22
Start loading time after surgery	
31A1	11.2 (7 ~ 16 days)
31A2	21.7 (18 ~ 35 days)
31A3	41.6 (35 ~ 72 days)
Harris hip score	85.6 (65 to 100 points)
Excellent	41 cases (25.15%)
Good	92 cases (56.44%)
Medium	26 cases (15.95%)
Poor	4 cases (2.45%)

**Table 3:** **Postoperative complications**

**Complications**	**Cases**
Cutout of the blade	3
Femoral head penetration of the blade	1
Thigh pain (outside)	16
Thigh pain (inside)	5

## Discussion

Intertrochanteric fractures often occur in older patients. According to some orthopedic surgeons, stable intertrochanteric fractures (Evans type I) can be effectively treated with conservative therapy and that surgical treatment should be reserved for unstable intertrochanteric fractures [1]. However, we believe that as long as the patient is expected to tolerate surgery according to their preoperative examination results, surgery should be performed even for stable undisplaced intertrochanteric fractures as soon as possible after the patient stands or sits up to avoid pressure sores and hypostatic pneumonia while reducing the risk of nonunion. Studies have shown that surgical treatment of intertrochanteric fractures is associated with a significantly lower mortality rate and higher quality of life than achieved with conservative treatment [2].

The dynamic hip screw is the representative nail–plate fixation system and is considered to be the gold standard treatment for intertrochanteric fractures. It has been widely used for this purpose, and years of clinical experience have demonstrated good clinical efficacy in stabilizing intertrochanteric fractures [3,4]. However, the dynamic hip screw does have some drawbacks, especially after application to unstable intertrochanteric fractures of the medial cortex because varus deformity and plate fracture can easily occur in such cases [5,6]. In elderly patients with osteoporosis, the hip screw-holding force is weaker than that in intramedullary fixation, which is more prone to rotation, hip screw cutting, and other complications [7,8]. Biomechanical studies have shown that because the mechanical axis of the intramedullary system is close to the center of the body, its mechanical properties are better than those of extramedullary fixation systems [9].

In 1998, the AO/ASIF organizational design and began applying PFN treatment to intertrochanteric fractures. Although the PFN overcomes many of the disadvantages of the conventional intramedullary nail, there have been many reports on the presence of the following complications of PFN in recent years: proximal screw cutting, intraoperative distal locking screw insertion difficulties, remote locking screw stress concentration caused by vegetation breaking into the matter, the Z effect, iliotibial tract irritation caused by anterior thigh pain, and others [10-13].

Because the PFN has some defects, the AO/ASIF improved the design of the PFN and introduced the PFNA system in 2003. The main change in the PFNA involves the end of the helical screw blade, which gradually increases the diameter to allow for compression of the bone around the femoral head, thereby stabilizing the femur and facilitating antirotation and compression [14,15]. Comparison of helical blades and ordinary lag screws for fixation of the femoral head by biomechanical methods in neck mechanic experiments has shown that the stability of reamers is obviously better than that of ordinary lag screws. The only drawback of the helical blade is that it cannot withstand fracture pressure as can ordinary lag screws; thus, surgeons should emphasize good fracture reduction [16].

Although the PFNA has a substantial number of advantages, many papers have reported complications during its clinical use, especially in Asian patients. First, the anatomical features of the PFNA do not match the femoral geometry of Asian patients. The standard length of a PFNA nail is about 200 mm, and the shortest is 170 mm. The stature of Asian patients is shorter than that of European patients, and the anterior arch of the physiological femoral curvature is relatively large; thus, the tops of the main staples of the PFNA easily tip over the anterior arch of the femoral curvature, resulting in femoral fracture [17]. If a full presurgical assessment is not performed to ensure that the diameter of the PFNA nail matches the patient’s anatomy, the risk of hip fracture during insertion may increase [18]. Second, the proximal nail of the PFNA may be longer in patients with a short stature so that walking induces friction between the nail and soft tissue of the thigh, causing pain [19]. Finally, the outer side wall of the proximal PFNA is circular, which will easily produce pressure on the lateral wall of the greater trochanter when the nail is easily inserted, resulting in bone damage and reset loss [20].

Because of the abovementioned shortcomings in the previous PFNA, The AO/ASIF improved the design and launched the PFNA-II. The PFNA-II exhibits some improvements in the design. First, the outer angle of the PFNA-II staples has been decreased from 6° to 5°, from the needle to the apex of the greater trochanter, to ensure that the canal is located in the middle of the distal nail and reducing the risk of the distal nail impacting the femur. Second, the distance of the proximal of the PFNA nail with the spiral blade and the tail cap of the spiral blade is longer, the proximal nail of the PFNA-II has been shortened to 45 mm, and the length of the helical screw end cap has been reduced to 2.5 mm. This reduces friction between the nail and soft tissue and decreases activity-induced hip pain, which arises from the friction of the nail with soft tissue. Third, the proximal end of the outer wall of the PFNA-II improved the nail from a round to a graphic design, thereby reducing stress caused by the nail impacting the medial femoral cortex and reducing the probability of fracture reduction loss when the PFNA nail is inserted into the femoral canal [21].

Although the PFNA-II has more advantages than the former design and has been significantly improved, some academic studies have shown that the femoral anterior arch of the PFNA-II does not match the anatomy of Asian patients. Even in the short-nail models of the PFNA-II (170 mm), only about 19.0% of the distal tip of the nail located in the central of needle canal, 74.7% located in the former, and 6.3% in the rear of the needle canal, not to mention the long nail. Contact irritation was present between the inappropriate nail tip position and the femoral cortex, which tended to increase patient discomfort and pain. The treatise’s conclusion is that both the long and short nails (nail length of ≤24 cm) require a curved design, which would be more suitable for the femoral anterior arch shape in Asian patients [22].

In conclusion, the PFNA-II has many advantages. First, the operation is simple and the operation time is short. In our clinic, the mean operative time using the PFNA-II was 42.5 min (range, 35–75 min), and major surgical procedures were completed in 30 min. Second, radiation exposure is low; the C-arm fluoroscopy of the PFNA-II was 2.5 times (2–4 times) in this clinical group. The main C-arm fluoroscopy focuses on whether the correct position be placed before inserting the guide pin and whether the location of the guide pin is correct after nail insertion. Third, the incidence of bleeding is low. Compared with the nail–plate system, the PFNA-II only exposes the tip of the greater trochanter, and most patients with osteoporosis do not require proximal reaming; therefore, the incision is small and less bleeding occurs during surgery. The volume of bleeding in our surgical group was only 67.5 mL (range, 42–150 mL). Although a few patients required a preoperative blood cell transfusion, no patients required an intraoperative or postoperative blood transfusion.

**Figure 1:**
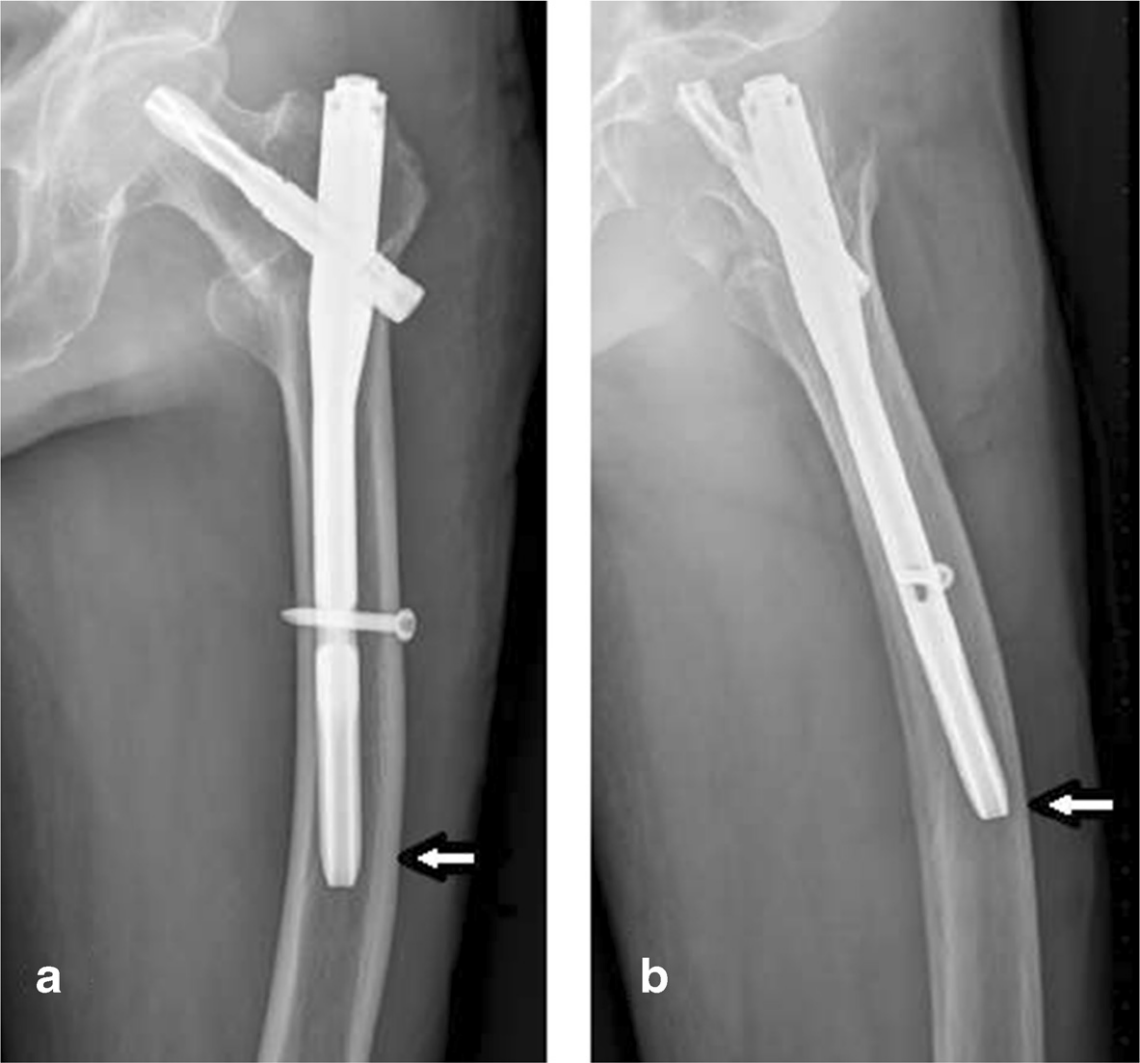
**X-ray shows the tip of the PFNA-II against the lateral wall in elderly patients with a larger natural anterior bow of the femur. (a)** Anteroposterior view. **(b)** Axial view.

The authors’ experience with the PFNA-II has shown that careful preoperative assessment is very important. Anteroposterior and lateral X-rays should be obtained preoperatively to estimate the size of the canal. For patients with a short stature or a large physiological femoral curve, the clinician should carefully consider whether or not to apply the PFNA-II. Because the short, narrow canal of most patients should undergo repeated reaming during surgery, the operative time will increase and blood loss will be significantly higher. In fact, most bleeding arose from the canal during the application of the PFNA-II to the treatment of fractures, and the amount of bleeding during repeated reaming was much higher than that in patients who did not undergo reaming. For patients with greater physiological femoral bending, the edge of the femur easily came into contact with the front or rear femoral cortex, causing severe pain (Figure 1).

**Figure 2:**
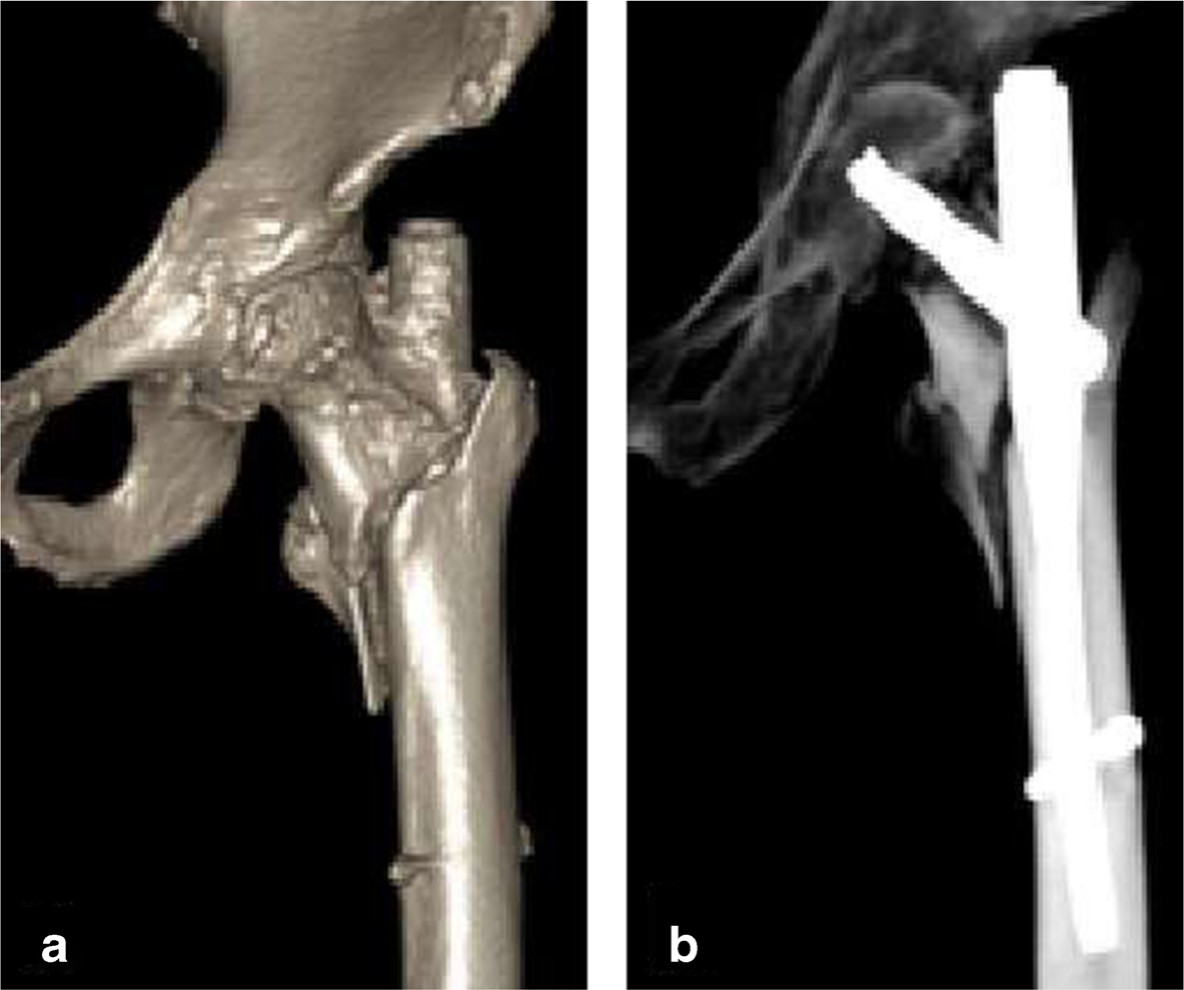
**In patients with a short stature, even after the use of a 170-mm PFNA nail, some portion of the long tail was still exposed outside of the greater trochanter, with hip joint activity easily leading to pain. (a, b)** Postoperative three-dimensional imaging.

In addition, compared with short-statured patients, the distance between the proximal aspect of the nail and the spiral blade still appeared to be relatively long. When the patient moved, the end of the nail exposed outside of the rotor readily came into contact with the iliotibial band, causing pain (Figure 2).

In this experiment, outer thigh pain was present in 16 patients (9.82%), and inner thigh pain (3.07%) was present in five patients (3.07%). Seven patients with severe pain experienced improvement after treatment with NSAIDs and physical therapy, and the remaining patients underwent no special treatment.

**Figure 3:**
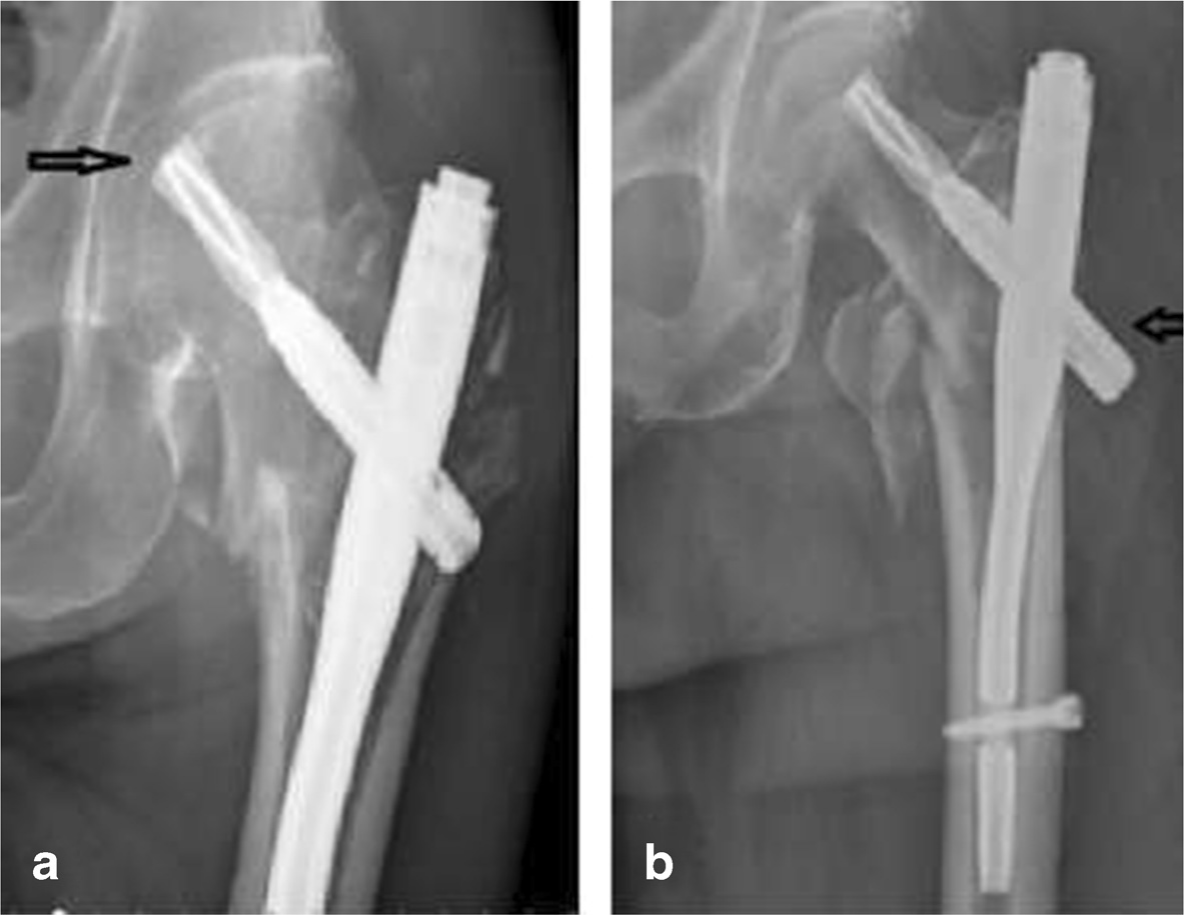
**Patients with severe osteoporosis may develop reamer cutting or piercing of the femoral head or back nails. (a)** Reamer cutting occurred in the presence of severe osteoporosis. **(b)** The entrance points of the PFNA nail needle are lateral deviation and the reamer exit.

Additionally, patients with severe osteoporosis may develop reamer cutting or piercing of the femoral head or back nails. One patient in this study developed reamer cutting and three developed piercing of the back nail; this prolonged the hospitalization time to allow for removal of the internal fixation device and allow for fracture healing (Figure 3).

We also believe that the most critical step of the surgical procedure is choosing the correct entry point before insertion of the PFNA-II needle. In general, the entry point is located at the top of the greater trochanter or slightly (about 3 mm) medially. An incorrect needle entry point will make nail insertion difficult. A violent insertion may lead to cortical rupture of the greater trochanter. Repeated reaming will increase the amount of bleeding and prolong the operative time. Determination of the position of the guide pin in the femoral neck is equally important. The best location of the needle is the lower one-third of the femoral neck, and it should be placed under anteroposterior fluoroscopy; when located in the center of the femoral neck, it should be placed under lateral fluoroscopy. If the position of the spiral blade in the femoral neck is too high, the risk of withdrawal or cutting of the spiral blade into the femoral head increases because of severe osteoporosis. When the spiral blade position is too low, it may damage the medial cortex of the intertrochanteric fossa and increase the risk of hip varus. After determining the position of the guide pin, it is crucial to measure its depth to determine the length of the spiral blade. Measurement of the depth of the needle guide should ensure that the sleeve is installed against the lateral femoral cortical bone; otherwise, the measurement result will be too long. A long spiral blade may increase the risk of cutting into the femoral head or significant pain caused by soft tissue friction with the end of the excessively long helical blade.

## Conclusions

This study shows that use of the PFNA-II to treat intertrochanteric fractures in elderly patients has the following advantages: a simple operation, few complications, and good clinical efficacy. The time of clinical treatment of PFNA-II was relatively short, and the clinical samples observed in clinical treatment were relatively small; the long-term complications remain unclear. Therefore, large-sample, multicenter studies are required.

## Abbreviations

PFNA-II: Asian proximal femur intramedullary nail antirotation system
PFNA: Proximal femoral nail antirotation system
ASA: American Society of Anesthesiologists
PFN: Proximal femoral nail
